# Microstructure and Mechanical Properties of B_4_C-HfB_2_-SiC Ceramic Composites Prepared by In Situ Reaction

**DOI:** 10.3390/ma19010082

**Published:** 2025-12-25

**Authors:** Langxiang Zhong, Qiang Liu, Chengmin Chen, Shuibao Liang, Zhihong Zhong

**Affiliations:** 1 School of Materials Science and Engineering, Hefei University of Technology, Hefei 230009, China; zhonglangxiang@hfut.edu.cn (L.Z.);; 2 Engineering Research Center of High Performance Copper Alloy Materials and Processing, Ministry of Education, Hefei University of Technology, Hefei 230009, China

**Keywords:** ceramics, B_4_C, HfSi_2_, microstructure, mechanical properties

## Abstract

B_4_C-HfB_2_-SiC (BCHS) ceramic composites were fabricated by in situ reaction with B_4_C and HfSi_2_ mixed powders through spark plasma sintering in this work. Effects of sintering temperature and sintering additive (HfSi_2_) content on the density, microstructure, and mechanical properties of the BCHS specimens were studied. The BCHS composite containing 15–30 vol.% HfSi_2_ reached over 98% theoretical density in the temperature range of 1600–1750 °C under the pressure of 50 MPa. The Vickers hardness, fracture toughness, and room temperature flexural strength of BCHS sintered at 1650 °C with 25 vol.% HfSi_2_ reached a maximum value of 31.3 GPa, 5.6 MPa·m^1/2^, and 573.9 MPa, respectively. The flexural strength was not decreased at elevated temperatures, and the flexural strength at temperatures of 400 °C, 600 °C, and 800 °C was evaluated to be 540.7 MPa, 518.8 MPa, and 586.9 MPa, respectively. Dense B_4_C-HfB_2_-SiC composites were fabricated by the spark plasma sintering process. The introduction of HfSi_2_ can significantly enhance the density and mechanical properties of B_4_C ceramic.

## 1. Introduction

Low-density ceramic, boron carbide (B_4_C), exhibits high hardness, good chemical stability, low thermal expansion, and strong capacity for neutron absorption [1,2], hence it is a preferred material for cutting tools, wear-resistant components, and nuclear control rods [3,4]. However, similar to many ceramic materials, the high melting point (2447 °C) and low self-diffusion coefficient of B_4_C make it difficult to achieve the full density material. Generally, B_4_C ceramic cannot achieve full densification below 2100 °C. In addition, the low mechanical properties of B_4_C, especially the low fracture toughness, are also the main obstacles in its engineering applications [5,6]. Hence, the density and mechanical properties of monolithic B_4_C ceramic are poor and need to be improved.

Recent studies indicated that the introduction of second phase, such as borides, carbides, oxides, and nitrides, effectively increased both the relative density and mechanical properties of monolithic B_4_C ceramic [7,8,9,10]. Huang et al. [11] prepared B_4_C-20% TiB_2_ ceramics through hot-pressing at 2000 °C and 25 MPa for 6 min. The resultant material showed a relative density of 99.9%, a bending strength of 650 MPa, and a fracture toughness of 3.2 MPa·m^1/2^. Yamada et al. [12] synthesized a B_4_C-20 mol% CrB_2_ composite by sintering at 2030 °C. This composite has a relative density of 98.1%, a fracture toughness of 3.7 MPa·m^1/2^, and a flexural strength of 525 MPa. In addition, Demirskyi et al. [13] realized similar improvements by incorporating 35% TaB_2_ into B_4_C. The resultant composite ceramics exhibited a microhardness of 26 GPa, a fracture toughness of 4.5 MPa·m^1/2^, and a flexural strength of 430 MPa. Metal oxides were also demonstrated to have a similar effect of improving the mechanical properties by the introduction of metal borides. The relative density, flexural strength, and microhardness of B4C-15 wt% ZrO_2_ mixed powder pressureless sintered at 2100 °C for 60 min were reported to be 96.7%, 364.5 MPa, and 34.2 GPa, respectively [14].

Similarly to other metallic borides, HfB_2_ also has high hardness, high melting point, and excellent electrical conductivity that can be compared with metallic conductors. Thus, the addition of HfB_2_ to the B_4_C should have a beneficial effect on the mechanical properties of B_4_C, and the incorporation of B_4_C and HfB_2_ is expected to produce a composite with high hardness and good electrical conductivity, which enables the B_4_C-HfB_2_ ceramic to be machined via electrical discharge machining (EDM). In addition, there is a significant mismatch in thermal expansion coefficients between HfB_2_ and B_4_C, which induces residual stresses among phases and crack deflection during the fracture of composites [15], thereby improving the fracture toughness of ceramics. However, both B_4_C and HfB_2_ have poor oxidation resistance. SiC exhibits exceptional oxidation resistance, chemical stability, and high hardness, making it the ideal phase for improving the comprehensive properties of B_4_C-HfB_2_ ceramic [7,16]. At elevated temperatures in an air atmosphere, SiC can react with oxygen to form a dense silicate glass layer, effectively inhibiting further oxygen diffusion and thus improving the oxidation resistance of B_4_C ceramics [9]. In addition, the oxidation product can seal the inherent defects, such as microcracks and pores, in ceramics, thereby enhancing the high-temperature mechanical properties of the ceramics to some extent [10]. Therefore, B_4_C-HfB_2_-SiC composite has the potential to possess good mechanical properties, machinability, and oxidation resistance.

Reactive sintering has been verified to be an effective way to obtain a composite with a fine and uniformly dispersed microstructure. B_4_C-HfB_2_-SiC (BCHS) ceramic composites were fabricated by in situ reaction with B_4_C and HfSi_2_ powder mixture through spark plasma sintering in this work. Effects of sintering temperature and sintering additive (HfSi_2_) content on the density, microstructure, and mechanical properties (hardness, flexural strength, and toughness) of the BCHS specimens were systematically studied. In addition, the high-temperature mechanical behavior of BCHS ceramic was characterized in an air atmosphere up to 800 °C, and excellent high-temperature flexural strength was achieved.

## 2. Experimental Procedures

Commercial boride carbide powder (97% purity, D50 is 3.5 μm, Mudanjiang Diamond Boron Carbide Co., Ltd., Mudanjiang, China) and HfSi_2_ powder (95% purity, D50 is 2 μm, Jinzhou Haixin Metal Materials Co., Ltd., Jinzhou, China) were used as raw materials. The chemical composition of the composites is shown in Table 1. B_4_C and HfSi_2_ powders were weighed and placed in a polyurethane jar with ethanol, and then ball-milled at 360 rpm for 12 h with a ball-to-powder ratio of 4:1 using a planetary mill (Changsha Miqi Instrument and Equipment Co., Ltd., Changsha, China). The slurry was dried at 80 °C for 12 h with a vacuum degree of ~100 Pa in a vacuum drying chamber, and then was sieved through a 200-mesh sieve. Subsequently, the mixed powder was loaded into a cylindrical graphite die with an inner diameter of 20 mm and then placed in a spark plasma sintering furnace with a pressure of 30 MPa. The sample was heated from room temperature to ~700 °C with a heating rate of 100 °C/min, held for 5 min for degassing, and then further heated with a pressure of 50 MPa. After reaching the target temperature (1600–1750 °C), the sample was sintered for 10 min, and then furnace-cooled to obtain the ceramic composite. The vacuum degree was ~20 Pa in the spark plasma sintering furnace.

The bulk density of the specimen was evaluated by the Archimedean method, and the sample was immersed in deionized water. The theoretical density was calculated based on the mixture rule using the X-ray diffraction (XRD) pattern of the as-sintered sample. The XRD analysis was performed to identify the phase composition of the raw powders and composites. XRD tests were carried out using an X-Ray diffractometer (D/MAX2500V, Cu target) produced by Rigaku, Tokyo, Japan. During the measurement process, the voltage and the current were 40 kV and 200 mA, respectively, and the scanning speed was set to 10°/min. Rietveld refinement for the quantitative phase analysis was performed via Jade 6.0 software. The microstructure of the raw powders and as-sintered composites were evaluated by scanning electron microscopy (SEM, Zeiss Sigma 500, Carl Zeiss AG, Jena, Germany) with an acceleration voltage range of 0.02–30 kV and a vacuum level of 10^−5^ Pa. Transmission electron microscopy (TEM, Tecnai G220, FEI, Hillsboro, OR, USA; accelerating voltage range: 20–200 kV, vacuum: ~10^−6^ Pa) equipped with a high-angle annular dark field (HAADF) detector and energy dispersive X-ray Spectroscopy (EDX) was also employed for microstructure and elemental distribution analysis. The TEM sample with a dimension of ~5 μm × ~5 μm was prepared by the focused ion beam (FIB) technique in the FIB-SEM device (Helios Nanolab 600i, FEI, Hillsboro, OR, USA).

The hardness was tested using a Vickers hardness tester (HVS-1000ZA, Shanghai Wanheng Precision Instruments Co., Ltd., Shanghai, China) with a load of 3 kg for 15 s dwell time; the hardness value was the average of 5 indentations. The specimen for the mechanical properties test was cut at room temperature by EDM with a size of 16 mm (length) × 2 mm (width) × 1.5 mm (thickness). The surfaces of the specimen were ground and polished with diamond slurries. The bending test loading rate was 0.5 mm/min, and the span was 14 mm in a universal mechanical testing machine (AGX-5KN, Shimadzu, Tokyo, Japan) at room temperature, 400 °C, 600 °C, and 800 °C in air. At least three specimens for each condition were tested, and the measurement accuracy was taken as the standard deviation. For the samples tested at elevated temperatures, the samples were heated with a heating rate of 10 °C/min to the test temperature in a furnace that was built into the universal mechanical testing machine and held for 10 min before loading. Fracture toughness was measured using the single-edge notched beam method on the same universal testing machine. The fracture toughness was measured by a single-edge notched beam test using the same samples of the bending test (notch depth = 0.5 mm) with a span of 14 mm and a loading rate of 0.05 mm/min. The fracture toughness was calculated from the following equation, and the values were determined based on the measurements of three specimens.

(1) $$K_{IC}=\frac{3PL\sqrt{a}}{2bh^{2}}[1.93-3.07(\frac{a}{h})+14.53{\left(\frac{a}{h}\right)}^{2}-25.07{\left(\frac{a}{h}\right)}^{3}+25.8{\left(\frac{a}{h}\right)}^{4}]$$

where $K_{IC}$ is the fracture toughness (MPa·m^1/2^), a is the notch depth (mm), b is the specimen width (mm), h is the sample height, P is the maximum load of the specimen at fracture (N), *L* is the span of the specimen (mm).

## 3. Results and Discussion

### 3.1. Phase Transition and Phase Identification

**Figure 1:**
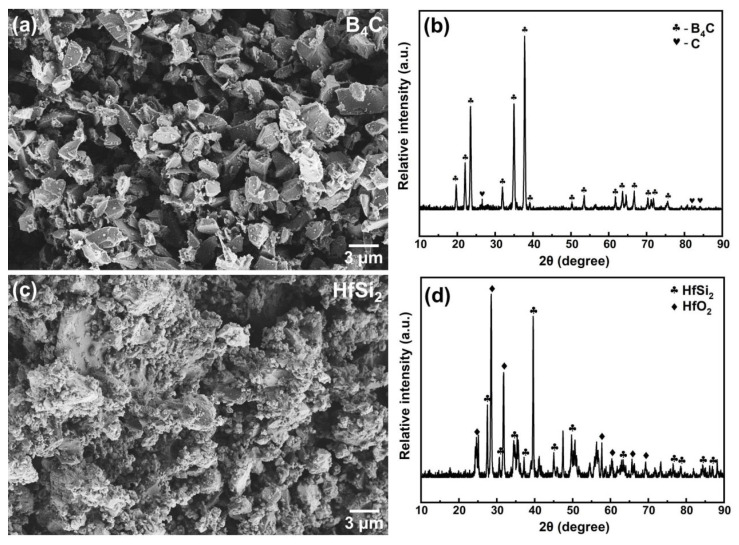
SEM image and XRD pattern of B_4_C and HfSi_2_ powder. (**a**) SEM image of B_4_C powder; (**b**) XRD pattern of B_4_C powder; (**c**) SEM image of HfSi_2_ powder; (**d**) XRD pattern of B_4_C powder.

**Figure 2:**
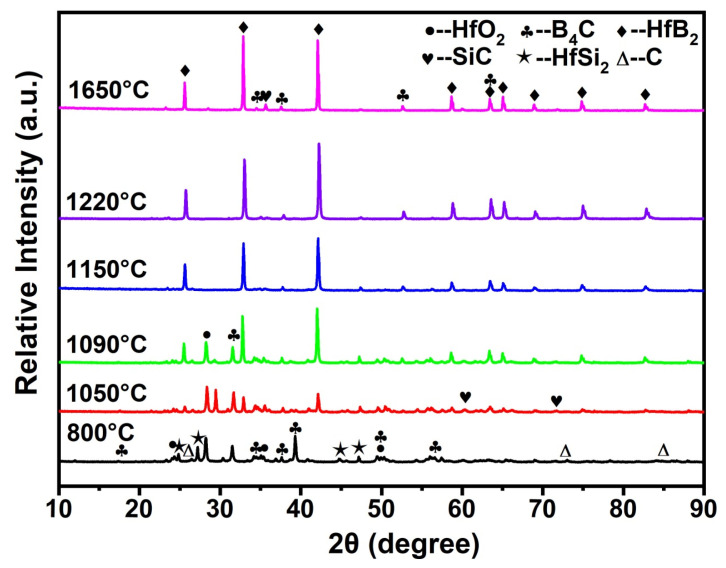
XRD patterns of ceramics with 30 vol.% HfSi_2_ sintered in the temperature range of 800–1650 °C.

Figure 1a,b shows the SEM image and XRD pattern of boride carbide powder. As observed, the boride carbide powder is wedge-shaped and has a smooth surface, and the crystal structure of boron carbide is B_4_C (PDF# 97-018-1128 card). Some free carbon (PDF# 97-018-8336 card) also existed in the raw material. Compared with B_4_C, the surface of HfSi_2_ powder is very rough, as shown in Figure 1c. The XRD pattern of HfSi_2_ (PDF# 97-001-6697 card) was also given in Figure 1d; it could be seen that HfO_2_ (PDF# 97-023-6776 card) also existed in the raw material. Figure 2 shows the XRD patterns of the samples with 30 vol.% HfSi_2_ sintered in the temperature range of 800–1650 °C. It shows that the phase compositions of the samples were changed with increasing temperature. For the sample sintered at 850 °C, it seemed that no apparent reaction occurred, as there was no new phase was detected. When the temperature increased to 1050 °C, HfB_2_ (PDF# 97-003-0422 card) and SiC (PDF# 97-061-8779 card) were found, suggesting that a reaction occurred in the mixed powders. Yet, the diffraction peaks of HfO_2_ and C also existed. As the temperature increased to 1090 °C, the phase composition remained the same, but the intensity of HfB_2_ dramatically increased. At higher temperatures of 1150 °C and above, HfO_2_ and HfSi_2_ were not detected, and the phase compositions of the composite were HfB_2_, SiC, and B_4_C. These changes indicate that HfSi_2_, HfO_2_, and C reacted with B_4_C to form HfB_2_ and SiC.

In this work, the possible way to form HfB_2_ is the reaction between HfSi_2_, B_4_C and C, or HfO_2_, B_4_C and C. Considering that HfO_2_ is located on the surface of HfSi_2_ particles, which is direct contact with the B_4_C and C, the reaction of Equation (2) may be advanced to Equation (3), although both reactions may occur spontaneously according to their Gibbs free energy (ΔG) calculated by the software (FactSage 8.4) at the temperature of 1050 °C (−8.36 for Equation (2) and −684.1 kJ/mol for Equation (3)). The reaction between the powder mixture of HfO_2_, B_4_C, and C was also confirmed by Sun et al. [17], who fabricated ceramic composites using HfO_2_, B_4_C, and C as raw powder. Similarly, the reaction between TiO_2_, B_4_C, and C to form TiB_2_ was also verified in reference [18]. The reaction of Equation (3) required the participation of free carbon. However, no additional C was added to the initial powder. The sources of C required in the reaction might be the C impurities in the B_4_C raw material (Figure 1a), the C diffusion of the graphite die [19], the pyrolysis of organic matter introduced in the mixing bottle (polyurethane) during the mixing process [20], and the C diffused from B_4_C [21].

2HfO_2_ + B_4_C + 3C → 2HfB_2_ + 4CO (2)

2HfSi_2_ + B_4_C + 3C → 2HfB_2_ + 4SiC (3)

**Figure 3:**
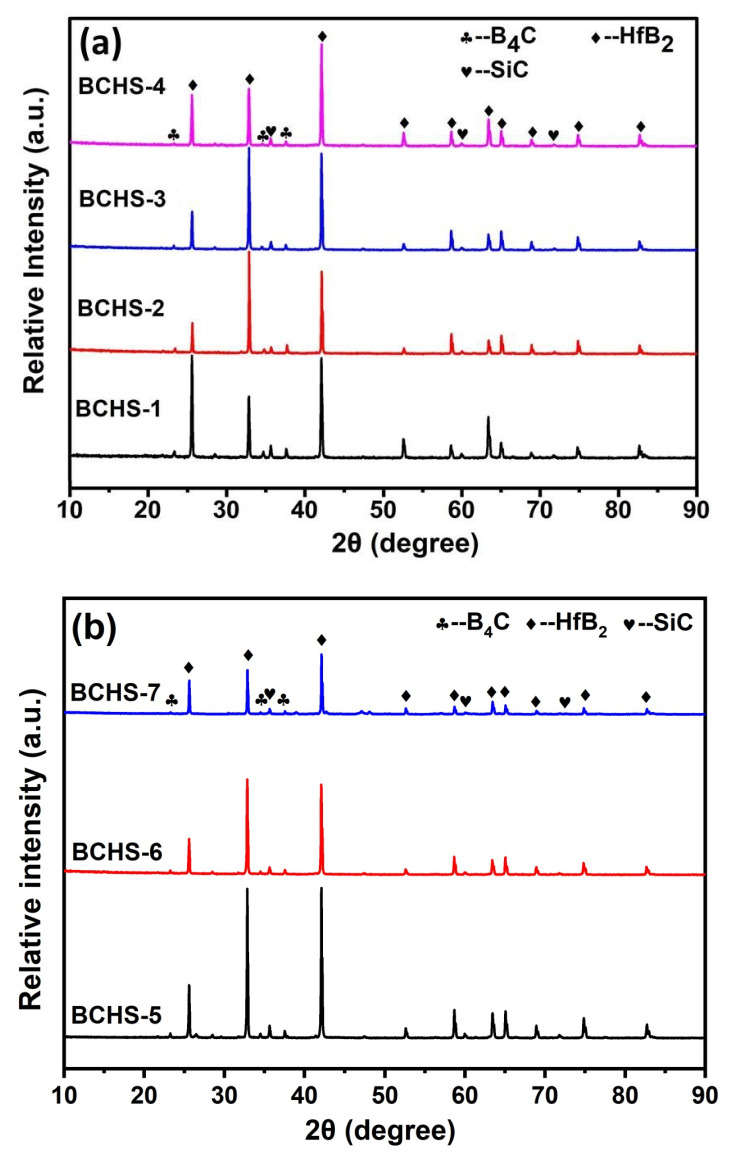
XRD pattern of ceramics sintered (**a**) with different amounts of HfSi_2_ and (**b**) at different sintering temperatures.

Figure 3a shows the phase compositions of samples with different amounts of HfSi_2_ sintered at 1650 °C. As observed, no characteristic peaks representing the phases of HfSi_2_, HfO_2_, and C appeared in the XRD patterns for each composition, implying that HfSi_2_, HfO_2_, and C reacted with B_4_C during sintering. It could be seen that the phase compositions of the samples were not changed with the increase in HfSi_2_ content, because the composites are composed of the same phases. Figure 3b shows the phase compositions of samples with 25 vol.% HfSi_2_ sintered in the temperature range of 1600–1750 °C. The sintering temperature up to 1750 °C did not have an obvious effect on the phase composition of the composites. This may be due to the stable thermophysical properties of B_4_C, SiC, and HfB_2_ at elevated temperatures. In addition, it is noted that there are slight differences in the relative intensities of the XRD diffraction peaks of HfB_2_ at the same diffraction position (e.g., BCHS-1 and BCHS-2), which may be attributed to the following reasons. First, the crystallinity of HfB_2_ formed by the reaction varies. Second, the uneven distribution of HfB_2_ in BCHS-1 induced defects such as stress, dislocations, and stacking faults inside the composite material, which in turn caused lattice distortion and ultimately led to changes in the diffraction peak intensity.

### 3.2. Relative Density

**Figure 4:**
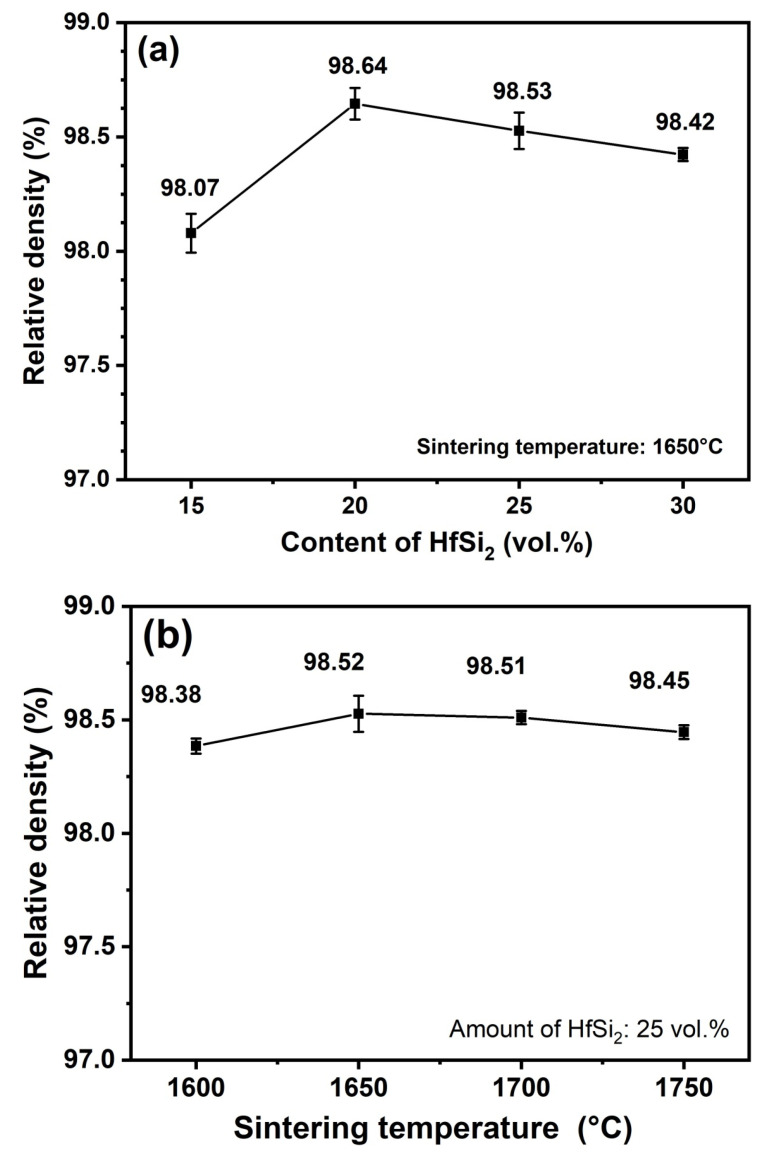
Relative density of ceramics sintered (**a**) with different amounts of HfSi_2_ and (**b**) at different sintering temperatures.

The theoretical density of B_4_C, HfB_2_, and SiC is 2.51g/cm^3^, 10.5g/cm^3^, 3.21g/cm^3^, respectively. The phase fraction and bulk density of the samples are given in Table 1. The theoretical density of the sample was calculated by the volume fraction weighted average method. Take the calculation of relative density for the sample BCHS-1 as an example; the theoretical density of BCHS-1 was calculated as 65.19% × 2.51 + 8.02% × 10.05 + 26.79% × 3.21 = 3.3383 g/cm^3^, and this value is divided by the bulk density (3.2739 g/cm^3^) to be 98.07% (the value of relative density). The relative density of other samples was calculated by the same method. Figure 4a shows the effect of HfSi_2_ content on the relative density of composite ceramics sintered at 1650 °C. It is shown that the relative densities of all specimens were higher than 98%, indicating that the composites had nearly full densification, and the incorporation of the HfSi_2_ sintering additive significantly improved the sintering behavior of the B_4_C ceramic. The relative densities of the composites sintered at 1650 °C increased first and then decreased with increasing HfSi_2_ content. The maximum relative density, 98.64%, was achieved for the specimen sintered with 20 vol.% HfSi_2_. The high density of the composite obtained by the power mixture in this work is attributed to the fact that during the sintering process, in situ reactions took place between the HfSi_2_, HfO_2_, C, and B_4_C, resulting in the formation of HfB_2_ and SiC with fine grains and strong bonding with each other. HfB_2_ and SiC could fill the pores among the powder particles through particle rearrangement under high pressure of 50 MPa. Additionally, B_4_C tends to develop structural defects during the in situ reaction process, forming non-stoichiometric B_4_C_1−x_ [21]. This also led to an acceleration of element diffusion, thereby facilitating the sintering densification process. Figure 4b shows the effect of HfSi_2_ content on the relative density of composite ceramics with 25 vol.% HfSi_2_ sintered in the temperature range of 1600–1750 °C. The relative densities of composite ceramics sintered at different temperatures were nearly identical, suggesting that the reaction and densification process of the composite was completed at 1600 °C.

### 3.3. Microstructure

**Figure 5:**
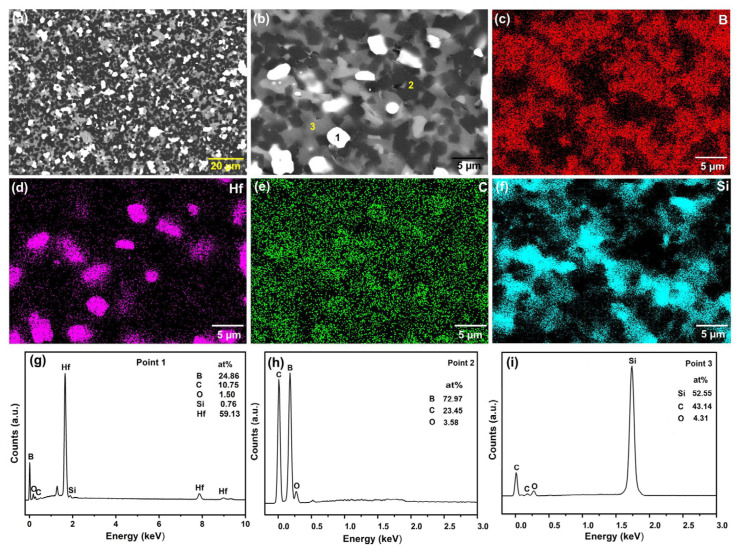
SEM image of BCHS-4 composite ceramic and EDS analysis. (**a**) SEM image of BCHS-4 composite ceramic; (**b**) SEM image of BCHS-4 composite ceramic; (**c**) B element distribution map; (**d**) Hf element distribution map; (**e**) C element distribution map; (**f**) Si element distribution map; (**g**) EDS analysis of Point 1; (**h**) EDS analysis of Point 2; (**i**) EDS analysis of Point 3.

Figure 5a shows the ceramic sintered with 30 vol.% HfSi_2_ at 1650 °C, and Figure 5b is the enlarged view of Figure 5a. It could be seen that the second phase was distributed homogeneously in the samples. Figure 5c–f shows the element distribution of B, Hf, C, and Si, respectively. As observed in these figures, the Hf element was concentrated in the white phase, and the Si element existed in the gray phase. Figure 5g–i shows the EDS point analysis of different phases in Figure 5b. The white phase was rich in Hf and B, the gray phase was abundant in Si and C, and the black phase contained primarily B and C. In addition, EDS analysis of these phases indicates that Si diffused into the matrix B_4_C, Si diffused into B_4_C, B diffused into SiC, which promoted mass transfer, enhancing the densification of ceramics. Combining the EDS analysis with XRD result shown in Figure 3, the different phases with different colors in Figure 5 are speculated as the white phase is HfB_2_, the gray phase is SiC, and the black phase is B_4_C.

**Figure 6:**
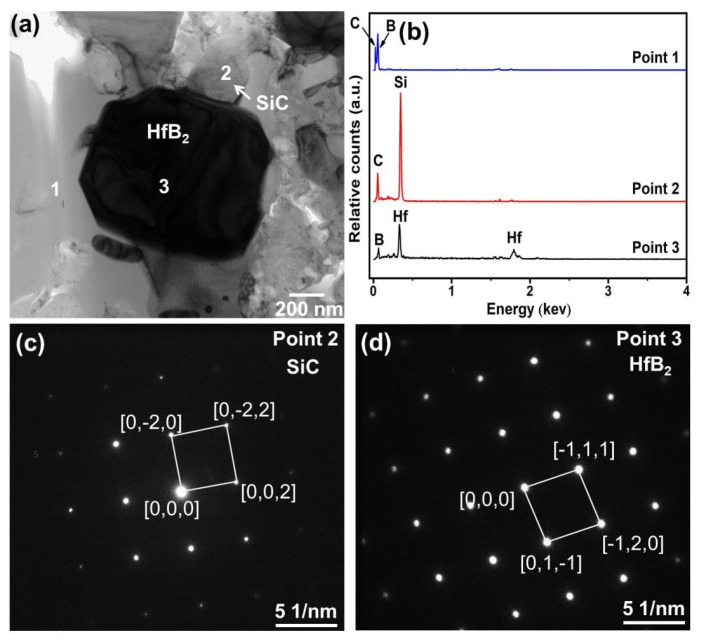
(**a**) TEM image of the BCHS-4 composite ceramic, (**b**) EDS spectrum at point 1, (**c**,**d**) diffraction spots at points 2 and 3.

In order to further identify the phases in the composite, TEM analysis was conducted, and Figure 6a presents the TEM image of the composite ceramic sample sintered with 30 vol.% HfSi_2_ at 1650 °C. As observed, the phases in the composite ceramic form tight bonds without any cracks or other defects. Figure 6b is the EDS spectrum analysis result of point 1, showing that the phase was composed of B and C. Figure 6c,d demonstrate the diffraction spot of point 2 and point 3, respectively. From the diffraction spots, the formation of SiC and HfB_2_ was confirmed again. Therefore, the ceramic consisted of B_4_C, SiC, and HfB_2_.

**Figure 7:**
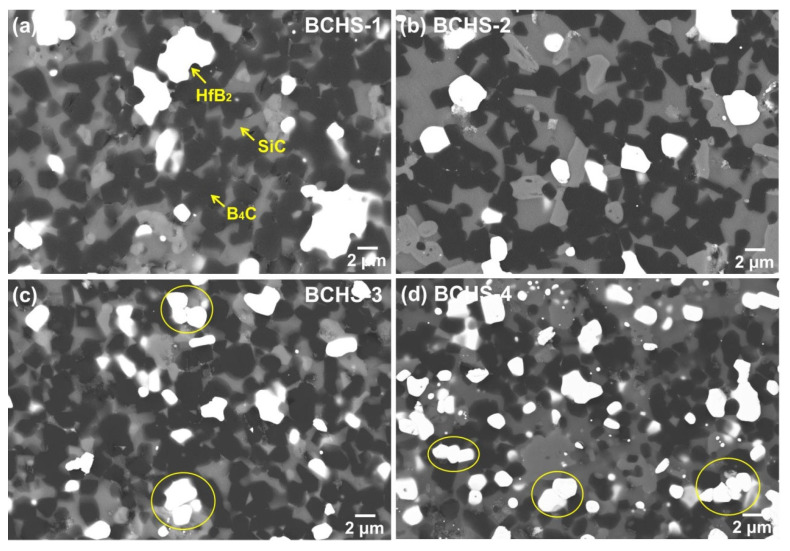
SEM-BSE image of the BCHS composite ceramics sintered at 1650 °C with different content of HfSi_2_: (**a**) 15 vol.%, (**b**) 20 vol.%, (**c**) 25 vol.%, (**d**) 30 vol.%.

Figure 7 presents the SEM-BSE images of the ceramics sintered at 1650 °C with different content of HfSi_2_. The microstructure of the composites was regular, and nearly no pores were observed, which is consistent with the results of the density measurement shown in Figure 4. A slight agglomeration of HfB_2_ was found in the specimen with 15 vol.% HfSi_2_, which is probably caused by the agglomerate of HfSi_2_ in the mixed powder, due to the inhomogeneous mixing or the presence of a large HfSi_2_ particle in the initial powder. As the HfSi_2_ content, the agglomeration was not found, and the HfB_2_ and SiC were distributed homogeneously in the samples. In addition, the densification of the specimen with 20 vol.% HfSi_2_ (Figure 7b) was higher than that with 15 vol.% HfSi_2_ (Figure 7a), which is consistent with the results of Figure 4. In addition, HfB_2_ had a tendency to agglomerate again for the samples sintered with 25 vol.% and 30 vol.% HfSi_2_, as indicated by the yellow circle in Figure 7c,d. The formation of connected HfB_2_ particles could be explained by two aspects. First, the amount of reacted formed HfB_2_ increased, which provided the possibility for the contact of HfB_2_ particles. Second, the formation of a liquid phase during sintering due to impurity oxides existed in the powders used. It is well known that during sintering, the liquid phase prefers to segregate the grain boundary of the matrix, which usually makes grain rearrangement at the initial stage of liquid phase formation. A similar result [21] was also reported in the ceramics of B_4_C sintered with MoSi_2_ as a sintering aid.

**Figure 8:**
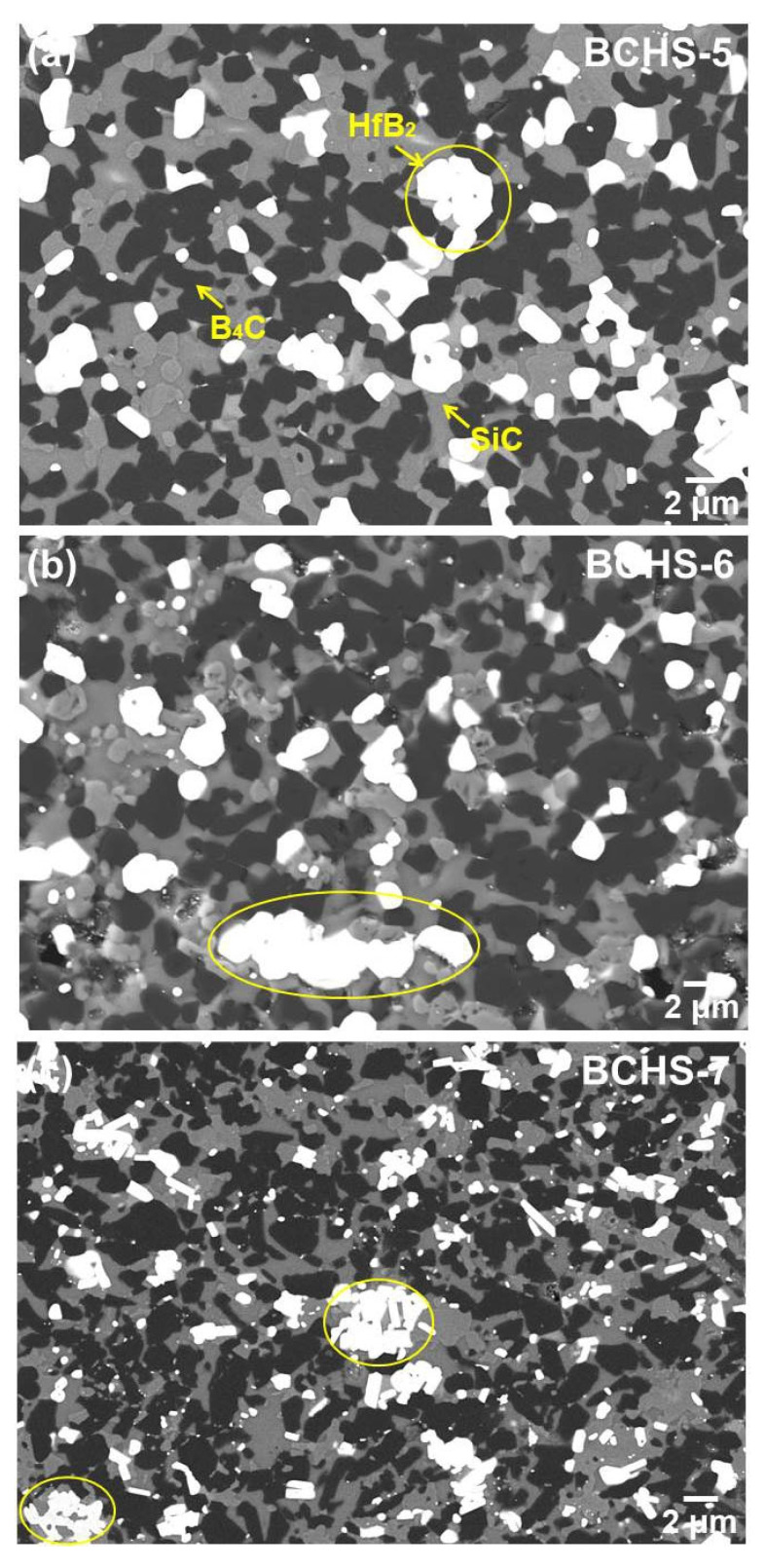
SEM-BSE image of the BCHS composite ceramics with 25 vol.% HfSi_2_ sintered at different sintering temperatures: (**a**) 1600 °C, (**b**) 1700 °C, (**c**) 1750 °C.

To investigate the effect of sintering temperature on the microstructural evaluation, SEM analysis for B_4_C-HfB_2_-SiC composite ceramics sample with 25 vol.% HfSi_2_ sintered at 1600–1750 °C was carried out, and the results are shown in Figure 8. Again, nearly no pores were observed in these samples, which is consistent with the results of relatively low density in Figure 4. Based on the results analysis above in Figure 7 and atomic weight differences in different phases, it can be concluded that the white phase is HfB_2_, the gray phase is SiC, and the black phase is B_4_C. A slight agglomerate of HfB_2_ was found in the specimen sintered at 1600 °C (as shown by the yellow circle in Figure 8a), probably owing to the agglomerate of HfSi_2_ in the powder. However, this agglomeration was not observed, and the second phase dispersed homogeneously in the samples sintered at 1650 °C. As increasing the temperature to 1700 °C and 1750 °C, the agglomerate of HfB_2_ occurred again. The reason for the agglomerate of HfB_2_ was analyzed before. It needs to be pointed out that the grain size of the composites was not dependent on the sintering temperature, since the grain size of the samples was similar. Hence, even if there was a liquid phase in the mixed powder during sintering, the amount of liquid phase should be limited.

### 3.4. Mechanical Properties

#### 3.4.1. Hardness

**Figure 9:**
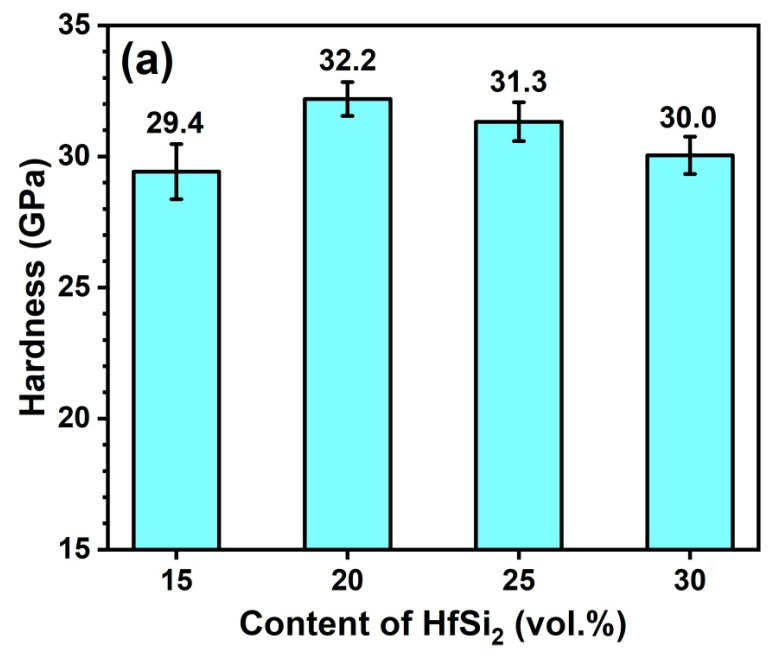

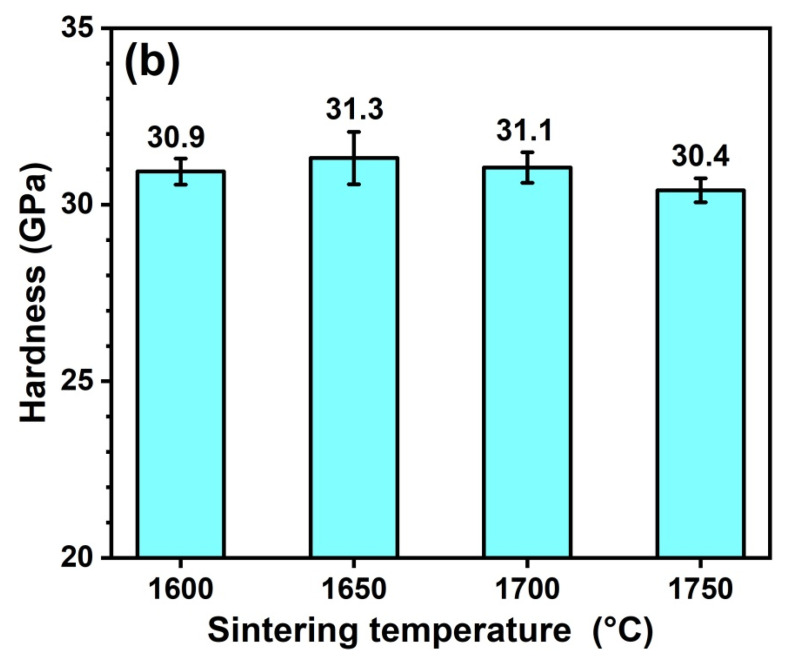
The hardness of BCHS composite ceramics as a function of (**a**) HfSi_2_ content and (**b**) sintering temperature.

Figure 9a shows the Vickers hardness of the sintered composites with different amounts of HfSi_2_. As observed, the hardness increased first and then decreased, with the different content of HfSi_2_ sintered at 1650 °C. The maximum hardness (32.2 GPa) was obtained for the sample sintered with 20 vol.% HfSi_2_. The hardness is closely associated with the grain size, relative density, and constituents of the ceramics. In this work, the growth of B_4_C grains was hindered by the in situ formed SiC and HfB_2_ particles, and the fine microstructure usually brings higher hardness. Considering that the grain size of composites sintered at 1650 °C was not dissimilar to each other (Figure 7), hence the relative density and the constituents of the ceramics may play a key role in the hardness. The hardness of BCHS-2 is higher than that of BCHS-1 because of its higher relative density, as shown in Figure 4. The hardness of B_4_C and SiC is 33 GPa [22] and 28–30 GPa [23], respectively. Both B_4_C and SiC have higher hardness than HfB_2_, with a hardness value of 18–22 GPa [22]. In addition, percolation theory shows that when the volume fraction of second phases is greater than 16%, percolation occurs [24], which can have a negative effect on improving hardness. This implies that sintering of B_4_C with the addition of HfSi2, which resulted in the formation of SiC and HfB_2_ (Figure 7), may not always increase hardness. Therefore, the incorporation of SiC and HfB_2_ to B_4_C-based ceramics can decrease hardness to some extent. Hence, the samples (BCHS-3 and BCHS-4) containing more volume fraction of SiC and HfB_2_ constituents have slightly lower hardness than the sample of BCHS-2 with lower second-phase content. In addition, the agglomerate of HfB_2_ in samples BCHS-3 and BCHS-4 is also detrimental to the increase in hardness. Figure 9b shows the hardness of the sintered composites with 25 vol.% HfSi_2_ at different temperatures. The hardness increased initially, followed by a slight decline, with the increase in sintering temperature. The maximum hardness value of 31.3 GPa was obtained for the composite ceramic sintered at 1650 °C. Considering that the relative density (Figure 4) and the volume fraction of second phases (SiC and HfB_2_) of the samples were almost the same, the agglomerate of HfB_2_ may also be responsible for the slight hardness decrease.

#### 3.4.2. Fracture Toughness

**Figure 10:**
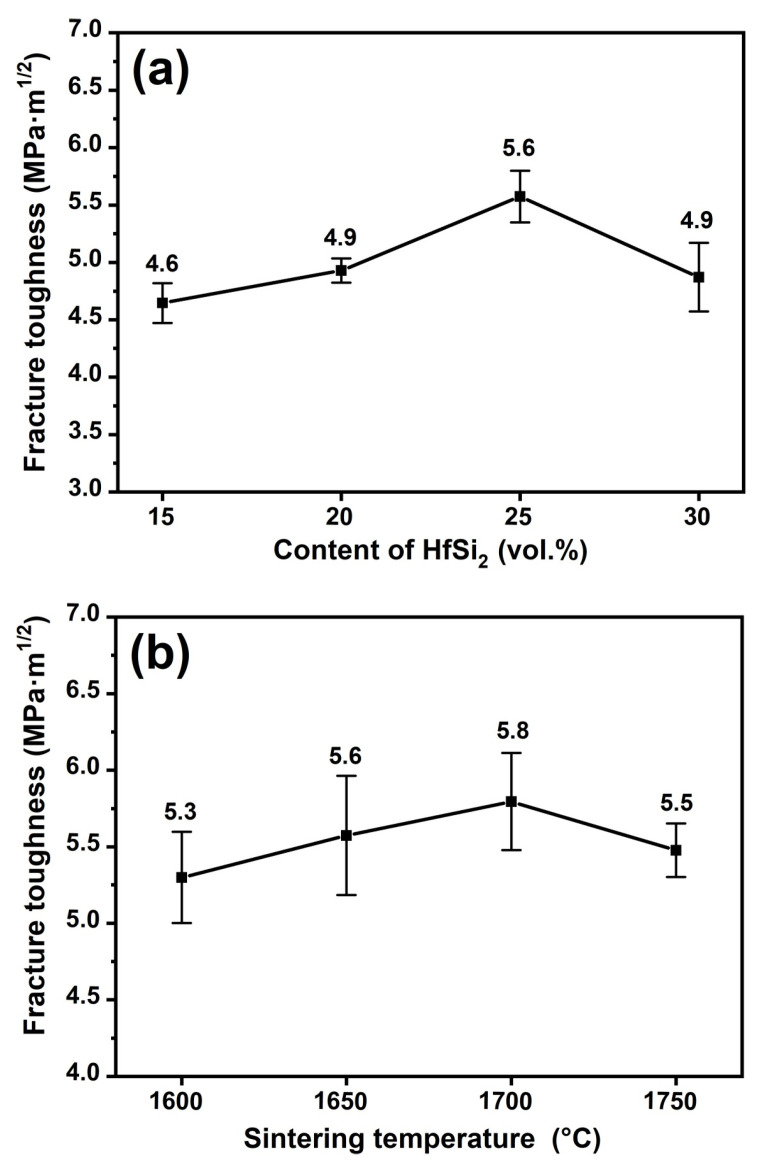
The fracture toughness of BCHS composite ceramics as a function of (**a**) HfSi_2_ content and (**b**) sintering temperature.

Figure 10 shows the relationship between the fracture toughness of the composite ceramic and the content of HfSi_2_ and the sintering temperature. As shown in Figure 10a, the fracture toughness increased with the content of HfSi_2_ and reached the maximum value of 5.6 GPa·m^1/2^ and then decreased to 4.9 MPa·m^1/2^. As presented in Figure 10b, a similar trend is observed for the fracture toughness of the composite ceramic sintered at different temperatures. With increasing the sintering temperature, the fracture toughness increased first and reached a maximum value of 5.8 MPa·m^1/2^ for the sample sintered at 1700 °C, and then decreased to 5.5 MPa·m^1/2^ for the sample sintered at 1750 °C.

**Figure 11:**
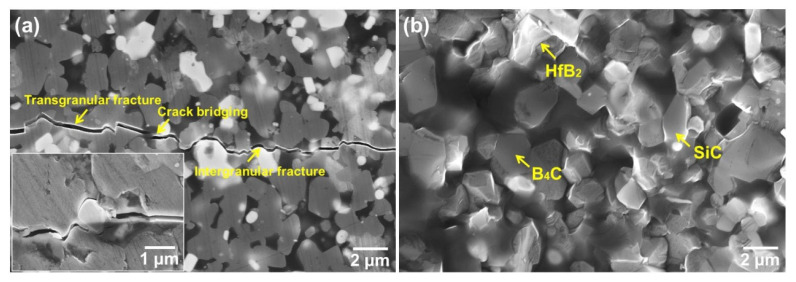
(**a**) Crack propagation paths of composite ceramics; (**b**) Fracture morphology of composite ceramics.

To identify the toughening mechanism, cracks were made by the hardness indenter on the surface of the sample with 15 vol.% HfSi_2_ sintered at 1650 °C, as shown in Figure 11a. It is shown that the crack propagation modes obtained by the indentation method were crack bridging, branching, and deflecting. Figure 11b is the fracture surface of ceramics with 15 vol.% HfSi_2_. Both transgranular and intergranular fractures are shown in Figure 11b, which is consistent with that observed in Figure 11a. The residual stresses intrinsically existed in the ceramic composites, originated from the CTE mismatch of constituents, are beneficial to the improvement of fracture toughness, because the residual stresses cause the crack to change its propagation direction and result in crack deflection and bridging [17]. Crack deflection and bridging due to the presence of residual stresses were reported in a previous study [21]. B_4_C grain fractured in a transgranular mode (Figure 11a). Cracks also propagated through the SiC grains, because the CTE of SiC (4.7 × 10^−6^ K^−1^) is similar to that of B_4_C (4.5 × 10^−6^ K^−1^). Hence, the presence of SiC was not the main factor for the increase in fracture toughness of the composites, although the fracture toughness of SiC is higher than that of B_4_C. The CTE of HfB_2_ (5.6 × 10^−6^ K^−1^) is higher than that of B_4_C and SiC; as cracks reached the HfB_2_ phase, the major fracture mode at B_4_C/HfB_2_ was transgranular fracture, as shown in Figure 11a.

With increasing the content of HfSi_2_, the amount of HfB_2_ and SiC in the composite increased. According to the above analysis, the fracture toughness could be enhanced with the increase in HfB_2_ amount, and hence the fracture toughness increased with the content of HfSi_2_ up to 25 vol.%. The fracture toughness slightly decreased for the sample with 30 vol.% HfSi_2_, probably due to the segregation of the HfB_2_ phase, which weakens the energy-consuming role of crack propagation. For the samples with 25 vol.% HfSi_2_ sintered at different temperatures, the difference in fracture toughness was insignificant, which could be attributed to the similar microstructure of these samples (Figure 8).

#### 3.4.3. Flexural Strength

**Figure 12:**
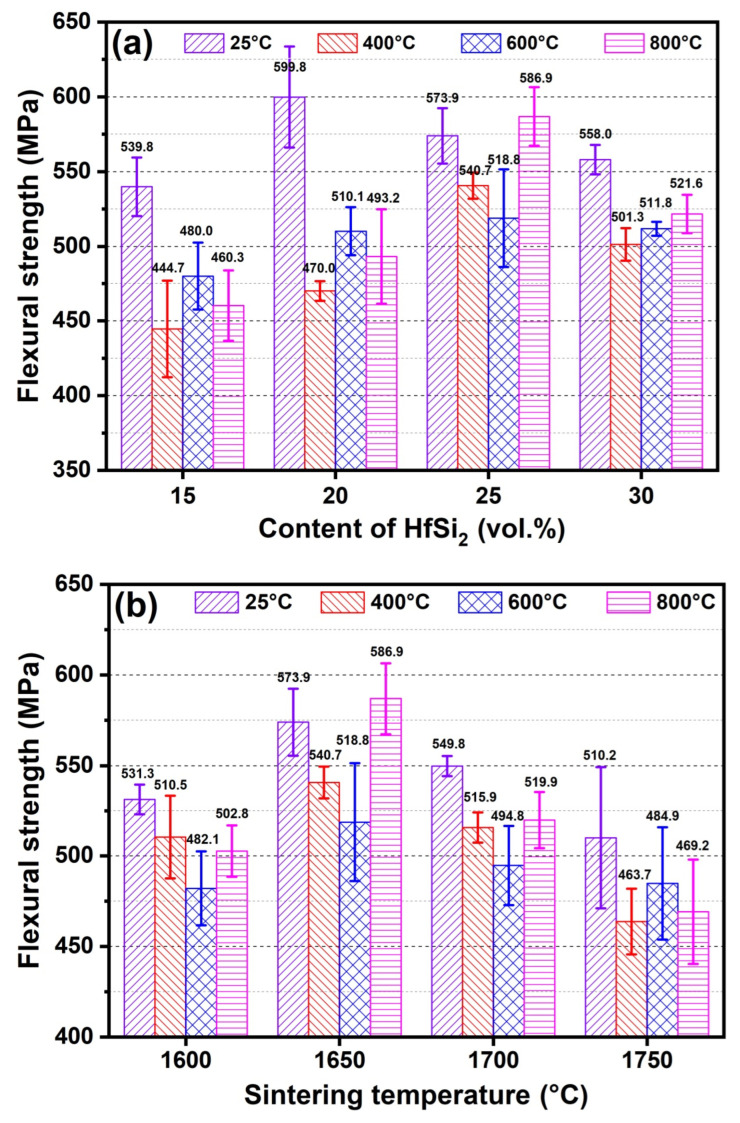
Flexural strength of BCHS composite ceramics sintered (**a**) with different content of HfSi_2_ and (**b**) at different sintering temperatures.

Figure 12a shows the flexural strength of composite samples sintered with different HfSi_2_ content. As shown in Figure 12a, at room temperature (RT), the fracture strength increased with the content of HfSi_2_ and reached the maximum value of 599.8 MPa, and then decreased to 558 MPa. At RT, the flexural strength of ceramic sintered with 20 vol.% HfSi_2_ (599.8 MPa) was higher than that of ceramic sintered with 15 vol.% HfSi_2_ (539.8 MPa). This is most likely due to the increased both relative density and volume fraction of reaction-formed HfB_2_ and SiC phases, which is beneficial for strength improvement. With the HfSi_2_ content increasing up to 30 vol.%, the slight decrease in flexural strength (558.0 MPa) could be attributed to the segregation of the HfB_2_ phase. With the testing temperature increasing, ceramics presented a decrease in flexural strength. At 400 °C, the flexural strength of samples sintered with 15 vol.% (444.7 MPa) and 20 vol.% HfSi_2_ (470.0 MPa) decreased obviously compared to that of RT, while the strength of samples sintered with 25 vol.% (540.7 MPa) and 30 vol.% HfSi_2_ (501.3 MPa) decreased slightly. In the temperature range of 400–800 °C, the flexural strength of ceramics remained at similar strength levels for each sample.

It is known that the high-temperature mechanical properties of non-oxide ceramics are greatly affected by their oxidation behavior. Thermodynamically, B_4_C-HfB_2_-SiC ceramics would be oxidized if they are exposed to air at high temperatures. The changes in flexural strength are attributed to the combination effects of oxidation damage and oxidation healing. The CTE differences between B_4_C, SiC, and HfB_2_ phases caused the different expanding behavior, which may provide more routes for oxygen transport at grain boundaries and lower the strength of the ceramic, thus the flexural strength of ceramics decreased at 400 °C. A similar result was reported in Ref. [16], where the flexural strength of 60 vol.% B_4_C-30 vol.% ZrB_2_-10 vol.% SiC ceramic decreased more sharply than that of 60 vol.% B_4_C-40 vol.% ZrB_2_ ceramic below 1000 °C was observed. B_4_C was reported to begin oxidizing at temperatures higher than 500 °C, and the oxidation of B_4_C led to mass gain due to the formation of B_2_O_3_, which has a low melting point (~450 °C). HfB_2_ begins to oxidize at ~700 °C and forms B_2_O_3_ and HfO_2_ in air, while SiC begins to oxidize at ~ 600°C to form SiO_2_. The oxidation of B_4_C, SiC, and HfB_2_ would heal the microstructure defects in the ceramics, resulting in the flexural strength of B_4_C-HfB_2_-SiC ceramics both at 600 °C and 800 °C decreased less than that at 400 °C.

Figure 12b illustrates the flexural strength of the composite ceramic containing 25 vol.% HfSi_2_ sintered at different sintering temperatures and tested at RT, 400 °C, 600 °C, and 800 °C. The bending strength at RT increased first and then decreased with the increase in sintering temperature, though these changes are minimal. Specifically, the sample sintered at 1750 °C exhibits the lowest flexural strength (510.2 MPa). The segregation of the HfB_2_ phase may be the reason for the strength decrease at 1750 °C. With the testing temperature increasing, ceramics presented a decrease in flexural strength. At 400 °C, the flexural strength of samples sintered at 1650 °C decreased slightly compared to that of RT (573.9 MPa vs. 540.7 MPa). Similarly to that observed in the samples sintered with different content of HfSi_2_, in the temperature range of 400–800 °C, the flexural strength of ceramics kept similar strength levels due to the oxidation of B_4_C, SiC, and HfB_2_ phases.

## 4. Conclusions

B_4_C-HfB_2_-SiC ceramic composites prepared by in situ reaction with B_4_C and HfSi_2_ mixed powder by spark plasma sintering were investigated in this work. The effect of HfSi_2_ content and sintering temperature on phase transformation, relative density, microstructure, and mechanical properties was studied. The conclusions are as follows:

(1) HfSi_2_ reacted with B_4_C to form HfB_2_ and SiC in the temperature range of 1600 °C to 1750 °C for the ceramics with different amounts (15–30 vol.%) of HfSi_2_. HfB_2_, SiC, and B4C are distributed uniformly in the ceramics.
(2) The formation of HfB_2_ and SiC improved the density and mechanical properties of B_4_C-HfB_2_-SiC composite ceramics. The relative density, Vickers hardness, fracture toughness, and flexural strength of ceramics sintered at 1650 °C with different content of HfSi_2_ increased first and then decreased with increasing the content of HfSi_2_. These properties for the ceramics containing 25 vol.% HfSi_2_ sintered at 1600–1750 °C also showed a similar trend.
(3) The optimal comprehensive mechanical properties of the B_4_C-HfB_2_-SiC composite ceramics were sintered at 1650 °C with 25 vol.% HfSi_2_. The relative density, hardness, fracture toughness, and flexural strength of ceramic sintered at 1650 °C with HfSi_2_ content of 25 vol.% was 99.5%, 31.3 GPa, 5.6 MPa·m^1/2^, and 573.9 MPa, respectively, at RT. The flexural strength at 400–800 °C decreased slightly compared to that tested at RT.

## Figures and Tables

**Table 1:** The nominal compositions of powder mixtures, the corresponding sintering temperature, and the phase fraction of samples.

Sample	B_4_C (vol.%)	HfSi_2_ (vol.%)	Sintering Temperature (°C)	Phase Fraction (vol.%)	Bulk Density (g/cm^3^)
BCHS-1	85	15	1650	B_4_C: 65.19; HfB_2_: 8.02; SiC: 26.79	3.2739
BCHS-2	80	20	1650	B_4_C: 58.24; HfB_2_: 11.01; SiC: 30.75	3.5559
BCHS-3	75	25	1650	B_4_C: 49.28; HfB_2_: 13.96; SiC: 36.76	3.8302
BCHS-4	70	30	1650	B_4_C:39.85; HfB_2_: 16.87; SiC: 43.28	4.0951
BCHS-5	75	25	1600	B_4_C: 50.51; HfB_2_: 14.14; SiC: 35.31	3.8253
BCHS-6	75	25	1700	B_4_C: 49.37; HfB_2_: 13.58; SiC: 37.05	3.8270
BCHS-7	75	25	1750	B_4_C: 47.83; HfB_2_: 14.05; SiC: 38.12	3.8246

## Data Availability

The original contributions presented in this study are included in the article. Further inquiries can be directed to the corresponding author.

## References

[1] Shan Q., Tian X., Xue Y., Hu J., You X., Wu B., Qian J., Wang Z. (2024). Initial damage behavior of Al_2_O_3_-modified SiCf/SiC-B_4_C composites after oxidation under wet atmosphere at 1200 °C. J. Am. Ceram. Soc..

[2] Zhang J., Zhang C., Zhang S., Zhang W. (2024). Mechanical properties and toughening mechanism of B_4_C-Al_2_O_3_ composite ceramics prepared by hot-press sintering. Ceram. Int..

[3] Takano M., Nishi T., Shirasu N. (2014). Characterization of solidified melt among materials of UO_2_ fuel and B_4_C control blade. J. Nucl. Sci. Technol..

[4] Chakraborty S., Debnath D., Mallick A.R., Das P.K. (2015). Mechanical, tribological, and thermal properties of hot-pressed ZrB_2_-B_4_C composite. Int. J. Appl. Ceram. Technol..

[5] Zhang X., Zhang Z., Wang W., Che H., Zhang X., Bai Y., Zhang L., Fu Z. (2017). Densification behaviour and mechanical properties of B_4_C–SiC intergranular/intragranular nanocomposites fabricated through spark plasma sintering assisted by mechanochemistry. Ceram. Int..

[6] Domnich V., Reynaud S., Haber R.A., Chhowalla M. (2011). Boron carbide: Structure, properties, and stability under stress. J. Am. Ceram. Soc..

[7] Balalan Z., Gulan F. (2019). Microstructure and mechanical properties of Cu-B_4_C and CuAl-B_4_C composites produced by hot pressing. Rare Met..

[8] Zou J., Huang S.G., Vanmeensel K., Zhang G., Vleugels J., Van der Biest O. (2013). Spark plasma sintering of superhard B_4_C-ZrB_2_ ceramics by carbide boronizing. J. Am. Ceram. Soc..

[9] Yamada S., Hirao K., Yamauchi Y., Kanzaki S. (2003). Mechanical and electrical properties of B_4_C-CrB_2_ ceramics fabricated by liquid phase sintering. Ceram. Int..

[10] Sousa J.M., Alves A.C., Toptan F., Ariza E., Guedes A. (2019). Corrosion and Tribocorrosion Behavior of Ti-B_4_C Composites Joined with TiCuNi Brazing Alloy. J. Mater. Eng. Perform..

[11] Huang S., Vanmeensel K., Vleugels J. (2014). In-situ Synthesis and densification of B_4_C-(Zr,Ti)B_2_ composites by pulsed electric current sintering. J. Chin. Ceram. Soc..

[12] Yamada S., Hirao K., Yamauchi Y., Kanzaki S. (2002). Densification behaviour and mechanical properties of pressureless-sintered B_4_C-CrB_2_ ceramics. J. Mater. Sci..

[13] Demirskyi D., Sakka Y. (2014). In situ fabrication of B_4_C-NbB_2_ eutectic composites by spark-plasma sintering. J. Am. Ceram. Soc..

[14] Lin X., Ai S., Feng Y., Gao D., Guo X., Liu Y., Xie B., Gong H., Zhang Y. (2017). Fabrication and properties of in-situ pressureless-sintered ZrB_2_/B_4_C composites. Ceram. Int..

[15] Tang J., Ji W., Xie J., Shi Y., Zhu Y., He Q., Wang W. (2020). Fine and High-performance B_6.5_C-TiB_2_-SiC-BN composite fabricated by reactive hot pressing via TiCN-B-Si Mixture. Ceram. Int..

[16] Qu Z., He R., Cheng X., Fang D. (2016). Fabrication and characterization of B_4_C-ZrB_2_-SiC ceramics with simultaneously improved high temperature strength and oxidation resistance up to 1600 °C. Ceram. Int..

[17] Sun W., Tian Y., Xu S., Liu L., Lu Y. (2023). Powder synthesis, densification, microstructure, and mechanical properties of HfB_2_-HfC ceramic Composites. Int. J. Appl. Ceram. Technol..

[18] Chenari P., Balak Z., Shahedifar V. (2025). Investigation of microstructure and mechanical properties of HfB_2_-HfC-TiC-B_4_C Composites. Mater. Chem. Phys..

[19] Ojalvo C., Guiberteau F., Ortiz A.L. (2019). Fabricating toughened super-hard B_4_C composites at lower temperature by transient liquid-phase assisted spark plasma sintering with MoSi_2_ additives. J. Eur. Ceram. Soc..

[20] He Q., Wang A., Liu C., Wang W., Wang H., Fu Z. (2018). Microstructures and mechanical properties of B_4_C-TiB_2_-SiC composites fabricated by ball milling and hot Pressing. J. Eur. Ceram. Soc..

[21] Wang S., Gao S., Xing P., Nie D., Yan S., Zhuang Y. (2019). Pressureless liquid-phase sintering of B_4_C with MoSi_2_ as a sintering aid. Ceram. Int..

[22] Feng B., Martin H.-P., Michaelis A. (2022). Preparation and characterization of B_4_C-HfB_2_ composites as material for high-temperature thermocouples. Crystals.

[23] Sun R., Wei X., Hu W., Ying P., Wu Y., Wang L., Chen S., Zhang X., Ma M., Yu D. (2022). Nanocrystalline cubic silicon carbide: A Route to Superhardness. Small.

[24] Leath P.L. (1976). Cluster size and boundary distribution near percolation Threshold. Phys. Rev. B.

